# Supplementary material for: From sequence to enzyme mechanism using multi-label machine learning
Source: BMC Bioinformatics. 2014 May 19;15:150. doi: 10.1186/1471-2105-15-150 (PMC4229970; doi:10.1186/1471-2105-15-150)
Supplement: Additional file 2 — Java code of ml2db. Additional file ml2db_code.tar.gz contains the Java source code to run the multi-label machine learning experiments and save the results to database. The code’s Javadoc is included. [file 1471-2105-15-150-S2.zip › additional file 2/ml2db/ml2db/doc/index-files/index-21.html]

U-Index


JavaScript is disabled on your browser.


- Overview
- Package
- Class
- Use
- Tree
- Deprecated
- Index
- Help

- Prev Letter
- Next Letter

- Frames
- No Frames

- All Classes

A B C D E F G H I J K L M N O P Q R S T U V W X 


## U

uncorrelatedLabels(int, double) - Method in class main.ml2db.mulan.mulanarff.Statistics
:   returns the indices of the labels whose phi coefficient values lie
    between -bound <= phi <= bound

UNDIRECTED\_ARROW - Static variable in class main.ml2db.mulan.mulanarff.NeighboursPrinter


UNIPROT\_DB\_PROPS - Static variable in class main.ml2db.ProjectParameters


UNKNOWN - Static variable in class main.ml2db.weka.AttributeFactory


UNLABELLED\_ARFF\_ID - Static variable in class main.ml2db.al.database.AlExperimentsTable
:   the table column to store the arff file id containing the unlabelled
    instances (in active learning, labelled instances to be "treated" as
    unlabelled)

UNLABELLED\_ATTRIBUTES - Static variable in class main.ml2db.al.database.AlEvaluationTable


UNLABELLED\_CLASSES - Static variable in class main.ml2db.al.database.AlEvaluationTable


UNLABELLED\_INSTANCES - Static variable in class main.ml2db.al.database.AlEvaluationTable


UNLABELLED\_ROLE - Static variable in class main.ml2db.al.utility.globaldistance.GlobalPositionStore


update(Instance) - Method in class main.ml2db.weka.JaccardDistanceStored


updateDistances(Instance) - Method in class main.ml2db.al.utility.globaldistance.GlobalDistanceMetric


updateDistances(Instance) - Method in class main.ml2db.al.utility.globaldistance.GlobalPositionStore


UTILITY\_FUNCTION - Static variable in class main.ml2db.al.database.AlExperimentsTable
:   a string describing the utility metric used to pick the next instance to
    be labelled

UTILITY\_FUNCTION - Static variable in class main.ml2db.al.database.UtilityTable
:   a string describing the utility metric used to pick the next instance to
    be labelled

UTILITY\_TABLE\_NAME - Static variable in class main.ml2db.al.database.UtilityTable
:   the name for the table to store the evaluation results

UtilityMetric - Class in main.ml2db.al.utility
:   A generic utility metric to pick the next (query) instance for active
    learning

UtilityMetric() - Constructor for class main.ml2db.al.utility.UtilityMetric


UtilityTable - Class in main.ml2db.al.database
:   Class

UtilityTable(DbManager) - Constructor for class main.ml2db.al.database.UtilityTable
:   the rank by utility metric of this instance (rank is from best to worst)

A B C D E F G H I J K L M N O P Q R S T U V W X

- Overview
- Package
- Class
- Use
- Tree
- Deprecated
- Index
- Help

- Prev Letter
- Next Letter

- Frames
- No Frames

- All Classes
